# From critic to inspirer: four profiles reveal the belief system and commitment to educational mission of medical academics

**DOI:** 10.1186/s12909-019-1665-0

**Published:** 2019-07-18

**Authors:** Marleen W. Ottenhoff- de Jonge, Roeland M. van der Rijst, Neil Gesundheit, Lianne N. van Staveren, Willem J. J. Assendelft, Friedo W. Dekker, Albert J. J. A. Scherpbier, Anneke W. M. Kramer

**Affiliations:** 1 Department of Public Health and Primary Care, Leiden University Medical Centre, Hippocratespad 21, Zone V7-P, PO Box 9600, 2300 RC Leiden, The Netherlands; 2 ICLON Leiden University Graduate School of Teaching, Leiden, The Netherlands; 3 Department of Medicine, Stanford University School of Medicine, Stanford, CA USA; 4 Department of Primary and Community Care, University Medical Centre St Radboud, Nijmegen, The Netherlands; 5 Centre for Innovation in Medical Education & Department of Clinical Epidemiology, Leiden University Medical Centre, Leiden, The Netherlands; 6 Faculty of Health, Medicine and Life Sciences, Maastricht University, Maastricht, The Netherlands

**Keywords:** Conceptions of teaching and learning, Faculty development, Medical education, Teacher attributes, Teacher identity, Teacher mission, Teacher profiles, Teacher qualities, Teaching beliefs

## Abstract

**Background:**

The educational beliefs of medical academics influence how they act in class and thus influence student learning. One component of these are beliefs academics hold about the qualities of teachers themselves. These teacher qualities range from behaviours and competencies to more personal attributes such as the teacher’s identity and mission. However, it is unclear what medical academics believe to be key teacher qualities. Therefore, this study explored the variety of medical academics’ beliefs about ‘teacher qualities’, aiming to identify and characterise profiles of academics with similar beliefs.

**Methods:**

We interviewed 26 expert academics from two medical schools to explore their beliefs about teacher qualities. A concentric onion-model focusing on teacher qualities was used to analyse and categorise the data deductively. Within each theme we developed subthemes inductively. To gain insight into the variety of beliefs we then clustered the participants into teacher profiles according to the themes. To better understand each of the profiles we carried out a quantitative study of the differences between profiles regarding subthemes, contextual and personal factors, and analysed statistical significance using Fisher’s exact- and Student’s t-tests for categorical and continuous data, respectively.

**Results:**

Four profiles of medical academics were identified, corresponding to the most central theme that each participant had reflected on: the ‘Inspirer’, ‘Role-model’, ‘Practitioner’, and ‘Critic’. The focus of the profiles varied from external constraining factors within the ‘Critic’ profile to affective personal qualities within the ‘Role-model’ and ‘Inspirer’ profiles. The profiles could be regarded as hierarchically ordered by inclusiveness. Educational institute was the only significant factor related to the profiles.

**Conclusions:**

Besides the relevance of affective teacher qualities, the ‘Inspirer’ profile demonstrates the importance of developing a clear mission as a teaching academic, centred around student learning and professional development. In our view, academics who inspire their students continue to be inspired themselves.

The practical implications are described for faculty development programmes, and for the potential value of using these profiles within medical schools. In the discourse on educational beliefs, the authors argue that more attention should be paid to affective qualities, in particular to explicating the educational mission of academics.

## Background

Most studies on educational beliefs until now have investigated the beliefs of academics from the perspective of teaching [1–10], without paying attention to personal aspects of teachers. Focusing on academics’ beliefs about teaching while leaving out their beliefs about *being* a teacher may result in too narrow an understanding of the phenomenon of academic teaching [11, 12], and does not take into account aspects such as the personal motivation of academics for teaching [12]. Thus, in order to obtain a more complete picture of all aspects relevant to beliefs about teaching, this study focuses on the beliefs that academics hold about being a teacher. Since the teaching beliefs of academics influence student learning and learning outcomes, it is important to obtain further insight into these beliefs [1–10].

First we will define some of the terms used in this study. Next, we will discuss the current state of knowledge regarding beliefs about teaching, and introduce Korthagen’s model which was developed to enable reflection on teachers’ beliefs about being a teacher and which we use to analyse our data.

Educational ‘beliefs’ refer to conceptions or convictions about aspects of education, such as teaching, learning, knowledge, students, or teacher qualities. The term ‘beliefs’ is generally used for those convictions that are formed early in life, are deeply rooted, and are harder to change than conceptions [13]. They are closely related to practice and can consist of both cognitive and affective aspects [14]. An example of a cognitive aspect is what teachers believe about desired learning outcomes, which may vary from recall of atomised information to a change in a student’s way of thinking. An example of an affective aspect is the role of interest and motivation in learning: some academics believe this to be teacher-initiated, while others believe it to be student-initiated [14]. Several terms are used to describe the personal aspects of being a teaching academic. We chose to use the term ‘teacher qualities’: an academic’s behaviours and competencies as well as the more personal attributes such as an academic’s identity and mission, both tangible and intangible, relevant to the functioning of a teaching academic. Academics in a medical context have not only an educational role, but other roles as well: those of clinician, researcher and/or administrator. Therefore, we have opted to describe medical teachers working in an academic context as ‘academics’.

The beliefs that academics hold about teaching and student learning have been investigated in a number of studies. While the outcomes of the studies differ in certain aspects, there is a consensus that teaching beliefs can be grouped broadly into teacher- or content-centred versus student-or learning centred teaching beliefs [8, 11, 12]. Central in a teacher-centred beliefs orientation is the focus of the academic towards knowledge transmission and the person of the academic and his/her teaching strategies, and in a student-centred teaching beliefs orientation towards the students’ conceptual understanding and the person of the students and their development [12].

A student-centred beliefs orientation is widely regarded as more developed than a teacher-centred orientation [8, 12]. Academics holding a teacher-centred orientation are likely to use a lecturing approach, even in small group sessions, whereas those with a student-centred orientation will use more interactive teaching methods. A student- centred orientation focuses on enhancing the learning of the student, even when teaching content. A prevalence of academics with a student-centred orientation leads to a deeper approach to learning, while a teacher-centred orientation leads to a more surface approach to learning [8].

Several factors, both contextual and personal, have been reported as being associated with teaching beliefs. Contextual factors described in the literature as being associated with teaching beliefs are level of the course [5], and discipline. The ‘hard’ disciplines [15], such as engineering or physics, are more often associated with a teacher-centred beliefs orientation, while in disciplines where attitudes and personal skills are more important, such as humanities or social sciences, student-centred orientations typically predominate [5, 16–20]. Other contextual factors reported are appreciation of teaching by the department [21], and the educational history and culture of the institute [22].

A number of personal factors is associated with teaching beliefs. In some studies female teachers [20, 22, 23] or experienced teachers [24, 25] are more likely to show a student-centred beliefs orientation, in another study this is not confirmed [26]. Other personal factors reported are educational role (teaching role only versus combined with educational management/ development and/or research) and type of educational task (lectures only versus mix) [22].

To better understand the variety of beliefs of individual teachers, several studies clustered teachers with similar beliefs into profiles [27–30]. By clustering, one can group together similar and homogeneous subsamples of people and analyse the characteristics of those participants that are clustered into the same group [31].

The literature provides two practical reasons why studies on teaching beliefs are important. A first reason is that it is important to investigate whether the teaching beliefs of medical academics are in alignment with the student-centred educational approach of their medical schools, because clearly, any curricular innovation that is inconsistent with the beliefs of those responsible for its execution is ‘doomed to fail’ [32]. A second reason to explore teaching beliefs is that these should be an important point of engagement in faculty development. The teaching beliefs that academics hold influence how they approach professional development, and how they benefit from it [33].

Insight into the beliefs of academics about teacher qualities can thus enhance the insight into the teaching beliefs of academics. However, in contrast to studies on beliefs about teaching, few studies on beliefs about teacher qualities are available within the context of higher education [11, 12, 34], let alone within the medical context [11]. While these studies drew attention to the person of the teacher when exploring academics’ teaching beliefs, they did not deliver a useful framework for our study. Because of our interest in obtaining a full image of all aspects relevant to beliefs about teacher qualities and their interrelatedness, we decided to opt for a different, more extensive model for the analysis of our data.

**Fig. 1:**
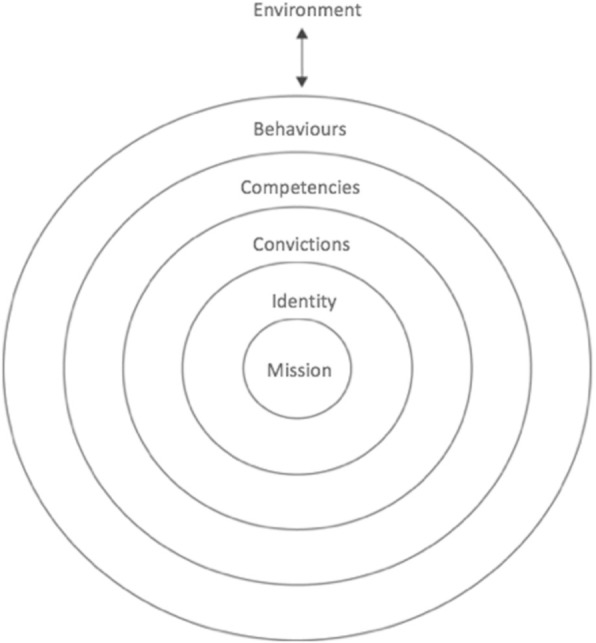
Concentric onion model of essential elements of teacher qualities (Korthagen 2004, adapted)

Within the field of teacher education Korthagen [35] developed a theoretical model to enable reflection on teacher qualities (Fig. 1), which integrates and relates different perspectives on teacher qualities in a holistic way. The model has been empirically evaluated in various international studies, in both medical and teacher education contexts [36–41].

This concentric onion model hypothesises that all levels of teacher qualities influence each other. These levels are: (1) Environment, (2) Behaviours, (3) Competencies, (4) Convictions, (5) Identity, and (6) Mission. The *environment*, the most peripheral level, can be defined as the context: persons, groups, and non-personal external factors that influence teacher qualities. The *behavioural* level refers to all concrete, observable behaviours or actions of the teacher. The level of *competencies* can be described as the integrated body of knowledge, skills and attitudes of the teacher. It represents a potential for behaviour and is not directly observable. The level of *convictions* refers to conceptions or perspectives teachers hold about learning, teaching, teachers or students. Korthagen refers to this level as the ‘beliefs’ level. Because of the way we defined the content and demarcation rules of this level, and to prevent confusion with our concept of beliefs about teacher qualities in the present study, we decided to name this level as the level of *convictions*. *Identity*, the next level, can be defined as the perspective teachers have on themselves, how they define themselves, how they perceive their professional role. Even though the focus is on the ‘self’, the perspective is relational, and largely determined by how relationships with significant others are viewed. Finally, the core level of the model, the level of *mission,* implies the intentions and goals a teacher pursues through teaching. What characterises the mission level is that it is focused on others, and on giving meaning to one’s own existence through contributions in a larger context. It has also been characterised as the ‘transpersonal’ level. Answering the question of *why* one teaches can lead to a reflection on the educational mission of the academic, whereas the question of *who* teaches can lead to further insight into the identity of the academic.

According to Korthagen, the process of a teacher’s professional development will be hindered when problems at a specific level are not tackled by progressing to a more central level, which underlines the importance of increasing our insight into these central levels of teacher qualities. For example, when academics reflect on their mission as a teacher they may discover that their professional inspiration mainly lies in their clinical work and much less in the relationship with students. A dialogue about what motivates these academics in their teaching might help them to become aware of their educational mission, which in turn may cause a shift in their perception of their professional identity and serve as an important motivator to challenge their educational convictions.

After analysing the beliefs about teacher qualities, we aim to explore whether profiles, clusters of academics with comparable beliefs, can be delineated. This will enable us to obtain a better understanding of why academics vary in their beliefs about teacher qualities, and to analyse which contextual and personal factors are associated with the academics that are clustered into the same profile.

By uncovering the variety in beliefs about teacher qualities through clustering into teacher profiles and exploring the characteristics of these profiles, we aim to deepen our understanding of the teacher, and in particular our understanding of aspects relevant to academics’ identity and mission, as these are at the core of the educational beliefs of academics and ultimately to their behaviour in class [35]. From a more practical perspective, the identified teacher profiles can provide input into how to shape faculty development programmes in medical schools with a student-centred curriculum.

Thus, our research questions are:

1. What is the variety of beliefs held by medical academics about teacher qualities?
2. How can the variety of beliefs about teacher qualities be clustered into profiles?
3. What is the relationship between the profiles and contextual (culture and organisation of the medical school, discipline) and personal factors (gender, teaching experience, teaching roles, educational tasks)?

## Methods

### Design and procedure

This exploratory, qualitative study consisted of four steps: the first step was largely deductive using Korthagen’s model as a template [42]. In the second step subthemes were developed inductively. The third step consisted of qualitatively clustering academics into profiles. The final step consisted of a quantitative analysis of the relationship with contextual and personal factors as well as subthemes.

The research is based on semi-structured interviews with medical academics, and is part of a larger research project exploring the longitudinal development of beliefs of medical academics about teaching, learning, knowledge, and teacher qualities, as, to date, the literature is not conclusive about if and how easily beliefs can change [8, 43, 44]. In this study we report on the outcomes of the baseline study executed in 2008–2010 regarding beliefs about teacher qualities. The medical academics invited to participate were all staff involved in a preclinical curriculum. The preclinical curriculum comprised both basic sciences and clinical topics. To investigate whether discipline is an influencing contextual factor, an equal number of academics teaching basic science and clinical topics were recruited as participants. To gain insight into the influence of the organisation we also interviewed academics at the highest administrative level. The study was conducted at two different medical schools, one in the Netherlands and one in the United States to obtain a deeper understanding of the influence of (national) culture and educational organisation as contextual factors. To exclude interpersonal variations during the interviews all the interviews were conducted by the first author. We started with the primary interview question of what makes a good teacher, and subsequently used unstructured follow-up questions to encourage participants to elaborate further, with the aim of developing an in-depth holistic image of the beliefs of the interviewees. Participants were asked to respond within the context of their preclinical teaching, to exclude differences in course level as an influencing contextual factor. We encouraged them to reflect on their teaching practice and to illustrate their responses with examples. The interviews lasted on average one hour and were audio-taped and transcribed verbatim by a research assistant. Prior to the interview the participant completed a brief survey to collect demographic data, including contextual factors (the name of the medical school, discipline) and personal factors (years of teaching experience, gender, educational roles, and type of educational tasks). The interview protocol as well as the survey questions were tested in a pilot study with three participants who did not participate in the main study. To address potential language issues we purposefully added a bilingual participant in the pilot study. Their comments were used to adjust and improve the instruments, including aspects related to language.

### Participants and setting

We estimated beforehand that 26 participants would suffice, as literature concerning sample size in qualitative research argues that if the participant sample is not too heterogeneous, sufficient (i.e. 88%) saturation will usually be reached with 12 participants [45]. Because we expected heterogeneity between the participants from the two medical schools and more homogeneity among the participants within each medical school, we decided to take 2 × 13 = 26 participants to be sure to include a sufficient number of participants. The participants in the respective medical schools were selected by a senior educator or sub-dean on the basis of their perceived expertise in teaching, based on student evaluations and obtained teaching awards. The rationale for this selection is that we were specifically interested in what the ‘best’, most respected -and therefore influential- teaching academics believe about teacher qualities. Of the 13 academics selected at each of the two medical schools involved, five taught basic science topics and five clinical topics and most of them were responsible for curriculum content; three were involved in educational administration. Of the 26 originally selected faculty, only one was not able to participate and was replaced by another faculty member who met the criteria.

The two medical schools were Stanford University School of Medicine (SUSM), California, USA, and Leiden University Medical Centre (LUMC), Leiden, The Netherlands. The interviews at SUSM took place in 2008, the interviews at LUMC in 2009/2010. Both medical schools are comparable in their emphasis on scientific education and had had their curricula redesigned in the decade prior to this study. At Stanford University School of Medicine a new preclinical curriculum was introduced in 2003. The hallmark of the new curriculum was the requirement for students to complete a Scholarly Concentration, a programme of study intended to integrate biomedical science, clinical medicine, and applied research in an area of a student’s personal interest. This component of the curriculum combined faculty-mentored research with structured coursework. The programme aimed to develop students’ skills essential for leadership in medicine and a lifelong commitment to cross-disciplinary investigation. The new curriculum also incorporated re-defined curriculum and increased time spent in direct patient-oriented learning. Classroom lectures were reduced from 30 h to 12–22 h per week [46]. At the time of the interviews, the medical curriculum of the Leiden University Medical Centre (LUMC) had been reformed in the decade before from a traditional teacher-centred curriculum towards a student-centred curriculum. Central educational characteristics of the curriculum were: a student-centred approach, active learning and emphasis on learning, thinking, and performing strategies rather than on only obtaining factual knowledge. The numbers of large group lectures were reduced, small group teaching sessions were introduced, and part of the study time was designated for self-study [47].

### Analysis

The analysis of the data followed the steps of the study design. The software programme Atlas-ti was used as the main supportive tool.

After immersion in the data, the first step was largely deductive: within the first twelve transcripts three team members (MO, LvS, RvdR) selected those text fragments that related to our first research question using the six levels of the onion model (Fig. 1) as initial themes, even though we kept the possibility for new themes open. These twelve transcripts were analysed independently to ensure data dependability. For each of the six themes the descriptions as formulated by Korthagen were the starting point; demarcation rules for each theme were fine-tuned during the analysis process to enable a consistent coding. We chose to code a text fragment within the *convictions* theme only if it could not be placed in one of the other five themes.

As a second step, since we uncovered a broad diversity of content within each of the themes, we developed, revised and refined subthemes inductively within each theme, using the procedure of constant comparison [48]. Descriptions and demarcation rules with respect to the subthemes were discussed within the research team, together with example text fragments. Within each theme, we reached ‘code saturation’ [49], meaning that no new subthemes emerged after we were about halfway through the analysis of all text fragments.

To calculate inter-rater agreement of the coding of themes and subthemes, the first author selected the text fragments of four not yet coded interviews–a total of 55 text fragments– as meaning units, which were coded independently by two team members (MO, LvS). This resulted in an inter-rater reliability of 0.87 (Cohen’s Kappa). Consensus was reached on the text fragments that were coded differently. The remaining transcripts were analysed by the first author, and discussed within the research team. After the coding procedure we re-read all transcripts again to ensure data confirmability, and to confirm ‘meaning’ saturation [49], that is we checked if we had harvested all new insights from the data.

During the third step, in order to answer our second research question we clustered the individual participants who shared similarities in themes qualitatively into teacher profiles. The theme that each participant emphasised as most relevant was leading for the clustering. To enhance the credibility and trustworthiness of this step in the analysis, the research team frequently gathered to discuss potential clustering, which had to be confirmed by the interview data before being accepted.

In the final step we analysed the participants in each profile quantitatively comparing them to all other participants with regard to the coded subthemes, which led to a richer picture of each of the profiles. In order to address the third research question the profiles were related to the contextual and personal factors. For this, we used a Fisher’s exact test to study differences regarding categorical data and a Student’s t-test for continuous data. IBM SPSS Statistics 20 was used.

### Ethical approval and consent to participate

This study was granted an ethics waiver by the Medical School Ethics Committee of the LUMC. The same committee advised that formal written informed consent was not required. All participants were invited by email or telephone by the first author, who emphasised that participation was voluntary and anonymous, and gave oral consent.

## Results

We decided to exclude one SUSM interview for further analysis, because the teacher-educator was responsible for the teach-the-teachers programme which we felt created a potential conflict of interest. Because of his educational expertise (he had obtained a PhD in education), he appeared to answer less from his own teaching experience and more from the theoretical framework that he used in his professional development programme.

In the first section of the results we will start with a description of the six themes. All six themes were adequately represented in the data (see number of text fragments per theme, Table 1), and data analysis did not generate new themes. Within each of the themes, subthemes were developed relating to teacher qualities, twenty subthemes in total, between two and four subthemes per theme (see Table 1). We will not here describe in detail the subthemes, but description of those subthemes that are relevant to the profiles will follow in the second section, in which the four profiles will be presented. After description of each of the profiles, including the relationships with contextual and personal factors as well as subthemes, in the last section we will describe our analysis of the relationship between the profiles.

**Table 1:** Themes of teacher qualities with corresponding subthemes, frequencies of text fragments, and examples

Theme (number of text fragments)	Subtheme	Frequency of text fragments (%)	Example (subtheme; case number)
Environment (85)	-related to non-personal factors	41	“*Too often I have the feeling that if I want to spend time and attention to teaching it has to be done in my own spare time, outside working hours.”* (related to administration; L01)
	-related to students	38	
	-related to administration	18	
	-related to peers	3	
Behaviours (56)	-related to students	41	“*they* [some teachers, ed.] *come in completely unprepared, and they haven’t thought about how they are going to convey the material to the students.”* (related to task; S01)
	-related to task	41	
	-related to self	18	
Competencies (55)	-related to students	36	*“Be someone who has the knowledge base. I think being on the same knowledge level as the student is not enough: you need to have the depth behind.” (*related to task as MD; L10)
	-related to task as educator	29	
	-related to task as MD	24	
	-related to self	11	
Convictions (22)	-regarding the teaching process: teacher- centred	32	“*A great teacher is of course also someone who stimulates the student asking relevant questions.*” (regarding the teaching process: student- centred; L04)
	-regarding the teaching process: student- centred	32	
	-regarding the teacher: not innate	23	
	-regarding the teacher: innate	13	
Identity (55)	-related to educational role	69	“*[...] be someone who is approachable and inviting for other opinions.*”*(*related to personal role; S04)
	-related to personal role	20	
	-related to patient-care role	11	
Mission (13)	-related to educational field	69	“*they* [good teachers, ed.] *can transmit their passion, […] are dedicated to their profession, they like sharing their enthusiasm.*” (related to medical field; S07)
	-related to medical field	31	

### Themes

The *environment* theme related to contextual factors which were considered as either constructive or obstructive to teacher functioning. The main distinction between the themes of *behaviours* and *competencies* lay in the fact that teacher behaviours would be directly observable in real time, and that academics usually phrased their beliefs about teacher behaviours in the present tense, while competencies would not be directly observable and were phrased as a potential for behaviour, like: ‘the ability to … ’ or ‘knowing how to...’. The *convictions* theme incorporated cognitive statements, describing convictions academics hold with regard to the teacher or regarding the process of learning and teaching. Coded text fragments were placed within this theme only *per exclusionem* (see methods)*.* The *identity* theme incorporated beliefs about the identity of academics; participants used phrases such as ‘Someone who is …. ’, ‘Being...’ and ‘Willingness to...’. The main distinction between the *identity* and *mission* theme was whether the academic focused on him/herself or on the student. The *mission* theme, the core theme, dealt with the academic’s deeper motivation for teaching which revolves around others and in which the student is central. This was in line with Korthagen’s definition of the mission level as a ‘transpersonal’ level [35]. Participants used terms like caring, sharing, helping, being dedicated, being passionate.

Even though the *environment*, *behaviours*, *competencies*, and *convictions* themes incorporated affective aspects, the content was mainly cognitive. Examples of these cognitive aspects are: ‘preparing a teaching session’ or ‘the ability to restructure’. Within the *identity* and *mission* themes this was just the opposite: the *identity* theme incorporated mainly affective aspects, while the *mission* theme contained exclusively affective aspects of teacher qualities, for example ‘sharing one’s passion for learning with the students’.

### Profiles

The clustering process generated four profiles. Each profile represents participants with shared beliefs about teacher qualities. Through discussions within the research team we concluded that the most central theme (corresponding to the level of the Korthagen model, see Fig. 1) that a participant had reflected on, was the most relevant to his/her beliefs. Therefore we clustered the profiles accordingly. The first profile included all the participants who had expressed beliefs related to the *mission* theme as their most central theme. The second consisted of all of the remaining participants who had reflected on the *identity* theme as their most central theme. The third contained all the remaining participants who had beliefs about *competencies* and *behaviours* themes as their most central theme. The fourth profile included the three remaining participants, all of whom had expressed beliefs related to the most outer theme of *environment*. The reason that we did not take the theme of convictions as a basis for a profile was because the four subthemes within this theme represent two mutually exclusive convictions, regarding the teacher and the teaching process, respectively. Thus, the academics who had expressed beliefs incorporated within the convictions theme did not all share similar beliefs.

Table 2 shows how the participants are divided among the profiles, and the themes within which each participant has reflected.

**Table 2:** Participants, demographical data, and themes, categorised per profile

Profile (n)	Case number	Faculty *	Gender (m/f)	Teaching experience (yrs)	Topic/ task	Mission**	Identity**	Convictions**	Competencies**	Behaviours**	Environment**
l. Inspirer (7)	S02	SUSM	m	15	Clinical	+	+	0	+	0	+
	S04	SUSM	f	10	Clinical	+	+	0	+	+	+
	S07	SUSM	m	15	Basic science	+	+	0	0	+	+
	S08	SUSM	f	15	Clinical	+	+	0	+	+	+
	S10	SUSM	f	19	Administrative	+	+	0	+	+	+
	S11	SUSM	m	38	Administrative	+	+	0	+	0	+
	S13	SUSM	m	25	Clinical	+	+	0	+	+	+
Mean				20							
II. Role model (10)	L01	LUMC	f	#	Administrative	0	+	0	+	+	+
	L04	LUMC	m	10	Clinical	0	+	+	+	+	+
	L05	LUMC	m	13	Basic science	0	+	0	+	+	+
	L06	LUMC	f	5	Clinical	0	+	0	+	+	+
	L08	LUMC	m	25	Clinical	0	+	+	0	+	+
	L10	LUMC	f	16	Basic science	0	+	0	+	+	+
	L11	LUMC	m	#	Administrative	0	+	0	+	+	+
	L12	LUMC	m	25	Basic science	0	+	+	+	+	+
	L13	LUMC	m	25	Administrative	0	+	+	+	+	+
	S01	SUSM	m	40	Basic science	0	+	+	0	+	+
Mean				20							
III. Practitioner (5)	L03	LUMC	m	20	Clinical	0	0	+	+	+	+
	L07	LUMC	m	16	Basic science	0	0	+	+	+	+
	L09	LUMC	m	25	Basic science	0	0	0	+	+	+
	S06	SUSM	m	19	Basic science	0	0	+	+	+	+
	S09	SUSM	f	35	Basic science	0	0	+	+	+	+
Mean				23							
IV. Critic (3)	L02	LUMC	m	10	Clinical	0	0	+	0	0	+
	S03	SUSM	m	24	Basic science	0	0	+	0	0	+
	S12	SUSM	m	32	Clinical	0	0	0	+	0	+
Mean				22							

*LUMC = Leiden University Medical Centre, The Netherlands; SUSM = Stanford University School of Medicine, USA

**0 = no codes in this theme; + = one of more codes in this theme

# = missing value

We related the four profiles to all 20 subthemes as well as to the contextual and personal factors to explore in which aspects the participants within a profile differed significantly from all other participants. This resulted in an in-depth description of each of the four profiles. We were unable to explore the roles of two of the four personal factors because they were the same for all participants, namely the factor ‘educational role’ as well as ‘type of educational task’. All our participants had not only a teaching role, but also roles in educational management/ development and/or research; and all participants were involved in both lecturing and small group teaching. Below we describe each profile. Where relevant, we include quotations from participants belonging to the profile concerned to clarify the core characteristics of the profile (main theme and subtheme labels are italicised).

#### Profile l: the ‘Inspirer’

The seven academics within this profile had in common that they all reflected on their *mission*. As the beliefs within the *mission* theme can be summarised as ‘inspiring students’, we gave this profile the name of ‘Inspirer’. Because these academics reflected on their mission as educator or as physician, we developed two subthemes: mission *related to educational field* and mission *related to medical field*. For example, they emphasised the importance of sharing one’s passion for learning (*mission/related to educational field*) or one’s passion for the medical profession (*mission/related to medical field*) with students. Some academics reflected on both:

*Enjoying your topic, enjoying your field,* ***caring about your students, that they learn. The number one thing is caring about the students*** (mission/related to educational field). *Having a lot of energy: people get more engaged by* ***people who are excited about what they are doing and part some of that energy*** (mission/related to medical field). *I think you can’t substitute anything for* ***caring that your students learn****.* ***The more you care, the better you are*** (mission/related to educational field). (S10)

Significantly, the academics within the ‘Inspirer’ profile more often reflected on the subtheme *identity/related to personal role* (*p* = .007) compared to the other profiles. Academics who were aware of their mission to support students’ learning and inspire students for the medical profession also emphasised the importance of an academic’s personal character qualities, such as being flexible, being receptive to feedback, being wise, acknowledging the limitations of one’s own knowledge:

*“What makes somebody a good teacher? I think* ***mostly caring about the students, caring that they learn something, that’s the first thing****”* (mission/related to educational field). *“Then* ***being someone who is approachable, who is wise and experienced but not intimidating or arrogant****”* (identity/related to personal role; S08).

Remarkably, the ‘Inspirer’ profile is the only profile in which academics did not express any belief within the *convictions* theme.

The Inspirer profile contained only academics from Stanford University School of Medicine (SUSM) (*p* = .002), while for other contextual or personal factors no significant difference was found as compared to the academics of the other profiles.

#### Profile II: the ‘Role model’

This profile contained ten academics who referred to *identity* as their most central theme. The three subthemes that emerged within the *identity* theme relate to the teacher role (*identity/related to educational role*), patient-care role (*identity/related to patient-care role*) and personal role (*identity/related to personal role*). We chose to characterise the academics of this profile as ‘Role models’.

In contrast to the academics of the ‘Inspirer’ profile who reflected on their personal role (*identity/related to personal role)*, this profile includes significantly more academics who refer to the subtheme *identity/related to educational role* (*p* = .018). Academics described the importance of being enthusiastic, being flexible in teaching methodology, being encouraging and supportive of the students and being interested in the development of the student.

*“Well, I think* ***a genuine interest in and engagement with the students****. You* ***should enjoy that and consider that important.*** *You* ***should also be concerned to see whether the student is developing in the direction that he should be developing in. That should give the teacher a thrill.”*** (identity/related to educational role; L01).

Other respondents stressed commitment as the main factor to being a good teacher: the willingness to teach and to improve one’s own teaching, to make learning fun or to work with students.

*“I think it’s just really* ***willingness to make the learning fun****. You know the students. They like teachers who allow them to learn something new”* (identity/related to educational role; S01).

With regard to contextual and personal factors, the ‘Role model’ profile included significantly more academics from LUMC (Leiden University Medical School) (*p* = .004).

#### Profile III: the ‘Practitioner’

The five academics in this profile all commented within the themes of *competencies* and *behaviours*. They did not refer to the more central themes of *mission* or *identity*. We called them the ‘Practitioners’, skilled professionals, who focus on the practice of education.

Looking at the subthemes, three academics expressed the *conviction* that being a good teacher is an *innate talent* (p = .004); no academic in the other profiles articulated such a conviction. Yet, all these three practitioners emphasised the importance of the appropriate competencies to being a good teacher.

All academics of the ‘Practitioner’ profile verbalised behaviours that are *related to students*; within this subtheme four distinctive aspects can be distinguished. Firstly, generating the students’ enthusiasm is considered a crucial element of teacher qualities.

*“You need to have* ***a motivating way of doing your presentation or coaching****. I think that's very important” (*competency/related to student/evoking enthusiasm; L03)

Secondly, relating to the student at an interpersonal level is considered important, for instance by creating a safe atmosphere, or by being available after class or by email.

Thirdly, respondents mentioned behaviours that should stimulate self-directed learning, such as encouraging students to ask questions or to actively participate during the session.

*“****Trying to let the students do most of the work, for that is the idea behind small group teaching. A teacher who just tells a story to the students [...], in the end that is not the purpose*** [of small group teaching]*”* (behaviours/related to student/stimulate self-directed learning; L09).

Fourthly, two respondents emphasised the importance of reflecting on group dynamics: picking up signals of what is going on in a group as well as accepting that within a group one should not try to please everybody.

*“You need to be able to put yourself in the position of the student as much as possible****.*** *It’s difficult, but you need* ***to have a feeling of balancing the needs of the different students****”* (competency related to student/dealing with the group; S06)

With regard to contextual and personal factors, the academics of the ‘Practitioner’ profile did not differ significantly from all other academics.

#### Profile IV: the ‘Critic’

This profile contained three academics who focused on the theme of *environment*. They did not make any references to *mission* or *identity*, nor did their comments refer to the *behaviours* theme. Two of the three respondents reflected on the *environment* and *beliefs* themes, while the third reflected on the themes of *environment* and *competencies*.

Compared to the other profiles the focus of the academics in this profile was on external factors that prevented them from being a good teacher: this was significant for factors attributed to their peers (*environment/peers*: *p* = .029), such as lack of recognition by colleagues that teaching is important.

*“****They*** *[other faculty, ed.]* ***don’t like to change things. Once they get it down they just want to keep doing it. Improvement is out of the realm of 90% of the faculty who are teaching. They’re busy people, it’s the last thing on their list, it has the lowest priority****”* (environment/related to peers; S03).

All three participants emphasised lack of time as an important constraining factor to good teaching. Two of the three participants mentioned factors caused by the administration of the faculty, for example, being responsible for sufficient finances, for explicit teaching rewards or for faculty development (a non-significant difference of *p* = .052). Because of this focus on external factors that need improvement, we named this profile the ‘Critic’ profile.

*“The most important is always* ***time and money*** (environment/related to non-personal factors)*, but let’s put those aside for a moment, because we’re not going to fix those. If someone felt that being a good teacher* ***brought rewards, some of them emotional, some of them promotional*** (environment/related to administration), *some of them* ***standing among their peers*** (environment/related to peers) *–* ***not everyone has to stand up and applaud every time you walk down the hall, but a sense that teaching is important, clinical care is important, research is important, administration is important, if you have any one of them not working, bad things happen****”*. (S12)

Within the *conviction* theme, two of the three respondents of the ‘Critic’ profile expressed a teacher-centred teaching orientation (*conviction/regarding the teaching process: teacher-centred*) (a non-significant difference of *p* = .057). However, it was not possible to determine the teaching orientation of the third respondent.

The academics of this profile did not differ significantly from all other academics regarding contextual and personal factors.

### Relationships between the profiles

The data suggested that the four teacher profiles were hierarchically ordered, the ‘Inspirer’ profile being the highest in hierarchy because of its inclusiveness. This is supported by the finding that academics of profiles higher in hierarchy reflected on an increasing number of themes, including those reflected on by academics of profiles lower in hierarchy. Thus, the main focus of the academics in the ‘Critic’ profile was on the *environment*. Within the ‘Practitioner’ profile, reflection was on *environment* as well as on teacher *behaviours* and *competencies*. Although the academics in the ‘Role model’ profile extended their elaborateness as far as teacher *identity* issues, academics in the ‘Inspirer’ profile reflected on all the themes, even on the core theme of teachers’ *mission* (see Table 2).

To illustrate the inclusive, elaborated beliefs of the ‘Inspirer’ profile we conclude with an example of an academic who verbalised his awareness of an effective teacher’s mission, identity, related competencies, and relevant environmental factors in a congruent way (coded text fragments in bold).

**Table:** 

S11:	(Sub)themes:
*“What makes someone an effective teacher is when* ***they are able to elevate the interest of the students*** *so that they*	(competence/related to students)
*desire to sustain in interaction and to learn more* ***; to really help inspire them*** *.[…] If you are really a great teacher*	(mission/educational field)
***you come to a topic with a great background of information and you are able to distil that in ways that present new challenges and opportunities***. *So I think that*	(competence/related to task as MD)
***great teachers bring out the best both in themselves*** *and*	(identity/related to personal role)
***in those that they are interacting with***.	(mission/educational field)
***Great learners are receptive to that and I think that a student could make -or not- a great teacher be effective***.”	(environment/related to students).

## Discussion

The educational beliefs of medical academics influence how they act as teacher and thus influence student learning. One component of these educational beliefs are the beliefs that academics hold about the qualities of teachers themselves. This study aimed to deepen our understanding of medical academics’ beliefs about these qualities. These teacher qualities range from behaviours and competencies to more personal attributes such as the identity and mission of the academic teacher.

The theoretical model of Korthagen (Fig. 1) proved to be a useful model to explore beliefs about teacher qualities. All six elements of the model could be uncovered, though some academics articulated their beliefs about teacher qualities more extensively than others. To better understand the variety of beliefs of individual academics we clustered them into four profiles; in each profile the academics focus on a specific subset of beliefs. We found the profiles to be hierarchically ordered based on the extent of inclusiveness of academics’ beliefs.

We will pay specific attention to the two most inclusive profiles, the ‘Role model’ and ‘Inspirer’ profiles, which focus on the two core levels of the onion model of teacher qualities (Fig. 1), a teacher’s identity and mission. The reason for this is that, as Korthagen (2004) presupposes, in the development of a teacher’s teaching beliefs the exploration of these two levels is crucial. Reflection on these two levels can help academics to become aware of their professional inspiration and their deeper motivation for teaching (their mission), as well as their professional identity as teacher. This can in turn motivate them to challenge their teaching beliefs and self-direct their own development. Development of the teaching beliefs of academics is relevant when implementing a curriculum innovation, since any change in curriculum design should go hand in hand with the development and change of the teaching beliefs of the academics who implement the innovation [32].

The medical academics in both the ‘Role model’ and the ‘Inspirer’ profiles acknowledge the importance of the academic’s identity. This identity is expressed in three distinct roles: the educational role, patient-care role and personal role. Two recent systematic reviews on doctor role modelling also conclude that the medical academic’s role encompasses not only the patient-care and educational domains but also the personal domain, and contains mostly affective attributes [50, 51]. In another study that compared academics’ beliefs about teacher qualities in preclinical versus clinical contexts, role modelling to enhance a medical student’s personal and professional development was only emphasised within the clinical context [11]. However, our findings show that also within the preclinical context, academics believe this teacher quality of role modelling to be important for student learning. Of the three above mentioned identity roles, the academics in the ‘Role model’ profile emphasise the educational role more than other academics. In contrast, the academics in the ‘Inspirer’ profile underline the personal role and characteristics, such as being wise, welcoming the opinions of others, being approachable. This emphasis is in alignment with their focus on the academic’s mission, which has been characterised by Korthagen (2004) as a ‘transpersonal’ level and should revolve around others, i.e. the students. Academics who are aware of their mission to contribute to the learning of the students realise that the best tool they have to achieve this is their own personality. A recent review concludes that an important aspect underlying the development of an academic’s identity is a ‘sense of commitment’. This ‘sense of commitment’ can be seen as a teacher’s mission, being described as ‘feeling a deep personal interest in teaching the next generation’ and having a ‘strong value in terms of caring for students’ [52]. In this review, which focused on the academic teacher’s identity, the relevance of explicating a teacher’s *mission* remained implicit. Yet, in our opinion, this finding relates the academic teacher’s identity to their mission and underscores the relevance of having an explicit educational mission. While several other studies within medical education have focused on the academic teacher’s identity, the academic’s mission has received little attention in the literature. Our study shows that medical academics within the most inclusive profile not only emphasise the academic’s identity but also the importance of developing a clear mission as a teaching academic. Spending time reflecting on why one teaches, on the deeper aims which one wants to achieve, can not only promote the development of an academic teacher’s identity but also can contribute to a deep personal involvement of the academic in student learning and ultimately in delivering the next generation of physicians. Thus, in the discourse on the academics’ teaching beliefs we recommend paying attention to awareness of identity, and in particular to educational mission.

In our findings the affective aspects of beliefs about teacher qualities, which are so prominently expressed within the ‘Role model’ and ‘Inspirer’ profiles, are articulated generically, transcending the context of pre-clinical teaching. Participants, for instance, emphasise their mission as the desire to bring out the best in the student, the willingness to make learning exciting, or the drive to improve one’s teaching. These teacher qualities will most likely not be limited to one particular context.

The findings of another study, executed in a non-medical context within higher education underscore the relevance of affective aspects, concluding that an important aspect of the academic’s beliefs about teacher qualities are teacher satisfaction and enjoyment [12]. This indicates that our conclusion that affective aspects are important also applies in contexts beyond those in which our study was executed.

Unlike other studies we found no significant relationship between the profiles and the academic’s discipline, gender, teaching experience, educational role or type of educational task. For the latter two factors this is related to the way the participants were selected, namely by perceived expertise: all our participants had multiple educational roles; and all participants were involved in both lecturing and small group teaching. For the other three factors discipline, gender, and teaching experience this is possibly because of the limited number of participants in this qualitative study. However, as with the findings of Jacobs [22], we did find a relationship with the medical school in which the academics were working: the ‘Inspirer’ profile consisted of exclusively SUSM teachers. This may be due to a difference in the medical school’s mission, or in the admissions policy for new staff. Another explanation could be a culture difference: in Dutch culture it may be less common to talk about one’s mission than in the American culture. Finally, it could also be related to professional development programmes and the teacher-educator responsible for this programme. Other research has emphasised the importance of the teacher-educator’s role in the educational development of academics [53]. At SUSM a faculty member provided teach-the-teacher seminars which emphasised the ability of an academic to inspire students to high achievement. A majority of the SUSM academics referred to his programme during their interview. No reference to any teacher-educator was made by LUMC academics. We presume that the relationship between academics and their students shows a parallel with the relationship between the teacher-educator and the academics in his teach-the-teacher seminars. In line with the findings of our study that academics within the ‘Inspirer’ profile have a drive to contribute to the learning of the students, the teacher-educator’s mission would be to contribute to the learning of the academics in his programme by helping them reflect on their teaching. The inspiration which he conveys may influence the articulated educational mission of the SUSM teachers.

Two other findings merit some comment. The first is that within the ‘Inspirer’ profile the convictions theme is absent. We presume that academics with more elaborate beliefs about teacher qualities are less likely to articulate these beliefs in general statements, but express their beliefs more explicitly. As the convictions theme is a theme *per exclusionem,* their articulated beliefs could be categorised in one of the other five themes. The second is that even though we did not explicitly explore the relationship between academics’ beliefs about teacher qualities and their teach*ing* beliefs orientation, in one of the profiles, the ‘Critic’ profile, the majority of academics expressed a teacher-centred teaching beliefs orientation. These academics mainly see themselves as responsible for the transfer of subject matter knowledge to their students, even though this is incongruent with the student-centred orientation of their medical school. Another study [27] also concluded that not all academics teaching in a student-centred curriculum embrace a student-centred teaching belief. Our findings raise the question whether there is a relationship between an individual academic’s teacher profile and the academic’s beliefs about teaching. Such a relationship is not unlikely, as in our findings the least inclusive profile, the ‘Critic’ profile, corresponds to a teacher-centred beliefs orientation, which is widely regarded as less developed than a student-centred orientation [8, 12].

### Limitations and implications for future research

This study was executed within the context of two medical schools. This may limit the immediate transferability of our findings to other medical schools. Secondly, almost ten years have passed between the data collection and the report of this study. However, we have no reasons to doubt that the four profiles derived from the beliefs of medical academics about teacher qualities are still current and valid. This is because we know that beliefs are relatively stable and hard to change [13]. Even though their distribution may change, we surmise that teacher teams will always include these four profiles. Thirdly, we purposefully selected the participants for perceived excellence and chose for a preclinical setting. Consequently, we have to be cautious in drawing conclusions from our findings for other contexts. Further study is needed to validate the profiles described in this study to investigate their characteristics in contexts beyond those of our study.

To gain more insight into the relationship between teaching behaviours of academics and their beliefs about teacher qualities, a study combining interview and observation could uncover (in)consistencies between individual medical academic’s beliefs and behaviours. Indeed, a recent mixed methods study showed a discrepancy between teacher beliefs and teacher behaviour [54].

Furthermore, we recommend future research to explore whether there is a relationship between the four teacher profiles on the one hand and a teacher- versus student-centred teaching beliefs orientation on the other hand.

Finally, our conclusion of the profiles’ hierarchical ordering does not imply that an individual academic will develop from a less to a more inclusive profile during his/her professional career. In the context of faculty development it would be relevant to study if academics show a shift of profile over time, and to explore the factors influencing such a shift.

### Practical implications

We anticipate three implications for faculty development in medical schools. The first is that the six levels of the onion model (Fig. 1) should all be taken into consideration when developing educational programmes for medical academics. At many medical schools, the focus of faculty development programmes is mainly on the levels of competencies and behaviours and not on the levels of convictions, identity, and mission [13], (p. 10). A second implication is that the twenty subthemes that we uncovered with regard to teacher qualities could be used as an innovative tool to encourage medical academics’ self-reflection. This could be incorporated into faculty development programmes, for instance by asking academics to reflect on the main question used for the interviews in this study: what makes someone a good teacher? Their answers could be compared to the subthemes, and this could help them to gain more insight into the variety of aspects important to being a good teacher. Third, the model of the four profiles of medical academics that we propose might serve to understand academics’ individual contributions within medical education: the ‘Inspirers’ can play an important role in inspiring both students and peers. In the larger context of the medical school this type of academic might play a significant role in the development of an educational mission with a focus on the student. The ‘Role model’ academics can contribute by being examples in role modelling: this is relevant for both students as well as for their academic peers. At the institutional level these academics might contribute by emphasising the importance of being a living example as teacher and as patient-caregiver. The importance of the ‘Practitioners’ is their practical emphasis; they can translate abstract concepts like inspiring ideas and role modelling into concrete behaviours. Within the medical school we suggest that the role of the ‘Critics’ might be to emphasise the importance of external factors such as sufficient time, priority, financial, and other resources being allocated to medical education.

Medical schools that are innovating towards student-centred curricula can benefit by considering these recommendations and incorporate them into their faculty development programmes. These programmes should not only pay attention to the teaching beliefs of the academics involved which need to be congruent with this student-centred orientation [27, 32]; they should also focus on all other aspects of teacher qualities, in particular on the academic’s identity and mission.

## Conclusions

Academics hold a variety of beliefs about teacher qualities. This study fills a gap by exploring this variety within a medical context. The concentric onion model of Korthagen (categorising teacher qualities from mission to environment) proved to be a useful model in the exploration of these beliefs. The variety of beliefs can be elucidated by the extent to which academics reflect on the levels of this model. Four hierarchically ordered profiles are identified, the ‘Inspirer’ profile being the highest in hierarchy, because it is the most elaborate profile and includes all levels of the model. The two least elaborate profiles predominantly focus on cognitive aspects of teacher qualities: external constraining factors, and practical competencies, respectively. The academics in the second most inclusive profile, the ‘Role model’ profile, extend their scope by emphasising the importance of being an example as a teaching academic to the students. Both the ‘Identity’ profile and the ‘Inspirer’ profile, the most inclusive profile, underline the relevance of the affective personal qualities of the teaching academic. The academics of the ‘Inspirer’ profile are unique in reflecting on their deeper motives when teaching. They demonstrate the importance of developing a clear mission as a teaching academic, centred around students’ learning and professional development. They emphasise their personal attributes as a main tool for accomplishing this mission. In our view, academics who inspire student learning and professional development continue to be inspired themselves.

The profiles are relevant for faculty development programmes, particularly in medical schools innovating towards student-centred curricula. An important conclusion from this study for the discourse on teaching beliefs is that in the development of teaching beliefs greater attention should be paid to the educational mission of medical academics.

## Data Availability

The datasets generated and/or analysed during the current study are not publicly available due to promised anonymity of the participants, but are available from the corresponding author on reasonable request and with permission of the participants in question. The generated codebook is available upon request.

## Abbreviations

LUMC: Leiden University Medical Centre
SUSM: Stanford University School of Medicine
